# Alumni Associations

**Published:** 1880-04

**Authors:** 


### EDITORIAL.

### ALUMNI ASSOCIATIONS.

THE commencements held by medical colleges at the close of the winter session, within the past month, have been supplemented generally by the assembling of graduates, old and new, in their alumni associations; the older members attracted hither from distant parts, and often from urgent professional work; the new recruits, with parchment just in hand, to pledge their troth to Alma Mater. In conformity with the annual custom, such a gathering took place in this city, in connection with the late successful commencement of the Buffalo Medical College. In view of the ability and reputation of the men sent forth by this institution, we have been led to expect something of real professional interest and value in its proceedings—some evidence of earnest scientific research, some additions to the stock of human knowledge in the special department assigned to the medical profession. If we say that in this respect we have been somewhat disappointed, it is not designed to cast unfavorable reflections, or to disparage either the men or the meeting. This leads to the suggestion that such associations, like other medical societies, would be doing better service to the profession in gathering from its members, now scattered in every state, and in contiguous as well as in distant provinces and countries, carefully collated facts, condensed from their experience and observation, than in scenes of banqueting, feasting and merriment.

It would indeed be an important step forward in the history of such organizations if their annual meetings could be made to develop an association of ideas, for the advancement of medical science alone. A wonderful impetus would thus be imparted to the medical philosophy of the present day, now depending rather upon the earnest labor of the few than upon the mass of the profession, who are willing to utilize all scientific research and investigations made by others, rather than to put forth an effort to contribute, even to a limited extent.

Many obstacles are in the way of securing methodical and consecutive work from such associations. As a rule they are wanting in the cohesiveness peculiar to like associations connected with departments of literature and the arts; but this deficiency is more than made up by the incentive to labor imparted to its votaries by the most humane of the learned professions. As now organized, they are simply auxilliaries in advancing the interests of medical colleges. We ask that their objects may be enlarged, and their field of labor broadened. The present faulty system of medical education also must be renovated. Medical schools should be encouraged by the profession at large, through individual and organized agencies, to adopt a higher standard and more extended study. The alumni of the respective medical colleges have a responsibility in this matter, which they may consistently share with their former teachers. Colleges are after all only representative agencies in the work of medical education. They can not be expected to rise above the sentiment of the profession and the community which fosters and sustains them. To mould this sentiment is a duty devolving upon the men duly commissioned by their authority. Let our alumni, therefore, seek a more complete organization, not only for original scientific research, but also to guard the great interests of the profession, of which they are for the time the custodians, in the matter of medical education.

